# Causal relationship among obesity and body fat distribution and epilepsy subtypes

**DOI:** 10.3389/fneur.2022.984824

**Published:** 2022-10-26

**Authors:** Kaiping Zhou, Huan Yang, Ruomeng Chen, Weiping Wang, Zhenzhen Qu

**Affiliations:** Key Laboratory of Neurology of Hebei Province, Department of Neurology, The Second Hospital of Hebei Medical University, Shijiazhuang, China

**Keywords:** obesity, fat distribution, epilepsy, Mendelian randomization analysis, causation

## Abstract

**Objective:**

The observational studies indicate an association between obesity and epilepsy, but it is unclear whether such an association responds to causality. The objective of this study was to determine the causal relationship between obesity and fat distribution and epilepsy subtypes based on waist circumference, hip circumference (HP), waist-hip ratio (WHR), and body mass index (BMI).

**Methods:**

A two-sample Mendelian randomization study was conducted separately for the four indicators of obesity and epilepsy and its seven subtypes, with reverse Mendelian randomization and multivariate Mendelian randomization for significant outcomes.

**Results:**

A two-sample Mendelian randomized analysis informed us that waist circumference was a risk factor for juvenile myoclonic epilepsy (beta = 0.0299, *P* = 4.60 × 10^−3^). The increase in hip circumference increased the risk of juvenile myoclonic epilepsy and epilepsy, with effect values of 0.0283 (*P* = 2.01 × 10^−3^) and 0.0928 (*P* = 1.40 × 10^−2^), respectively. Furthermore, children with a higher BMI exhibit a higher risk of epilepsy (beta = 0.0148 *P* = 1.05 × 10^−3^). The reverse Mendelian randomization study revealed that childhood absence epilepsy increased its BMI (beta = 0.8980, *P* = 7.52 × 10^−7^), and juvenile myoclonic epilepsy increased its waist circumference (beta = 0.7322, *P* = 3.26 × 10^−2^). Multivariate Mendelian randomization revealed that an increase in hip circumference and waist-hip ratio increased the risk of juvenile myoclonic epilepsy, with an effect value of 0.1051 (*P* = 9.75 × 10^−4^) and 0.1430 (*P* = 3.99 × 10^−3^), respectively, while an increase in BMI and waist circumference instead decreased their risk, with effect values of −0.0951 (*P* = 3.14 × 10^−2^) and−0.0541 (*P* = 1.71 × 10^−2^). In contrast, multivariate Mendelian randomization for childhood absence epilepsy and epilepsy did not identify any independent risk factors.

**Significance:**

Our findings provide novel evidence in favor of obesity as a risk factor for epilepsy and waist circumference as a risk factor for juvenile myoclonic epilepsy. Increased hip circumference confers an elevated risk of juvenile myoclonic epilepsy and epilepsy (all documented cases), and a high BMI increases the risk of childhood absence epilepsy. With this, new insights are provided into the energy metabolism of epilepsy, which supports further nutritional interventions and the search for new therapeutic targets.

## Introduction

**Figure 1:**
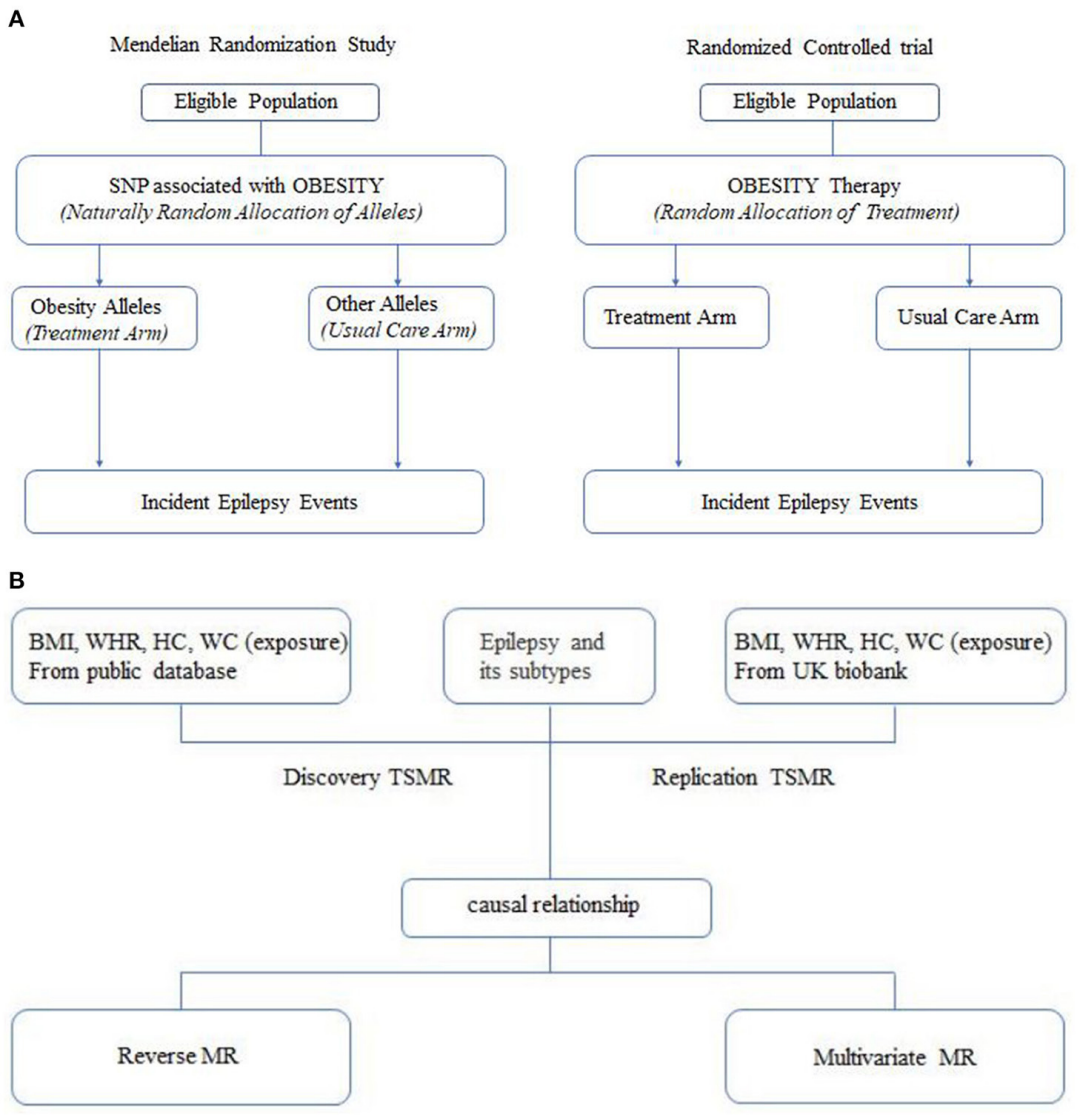
**(A)** Analogy between a mendelian randomization study and a randomized trial. **(B)** Flow diagram.

Epilepsy is one of the most prevalent neurological diseases in clinical practice, with more than 70 million people affected around the world (1). This diseases is of great importance for the economic health of many countries. An estimated 25–30% of patients suffer from uncontrolled seizures, although on medication (2). Comorbidities of epilepsy are also perceived as a conundrum in the treatment process. Therefore, more studies are required to identify potential causal risk factors that can help guide prevention efforts. Typically, the gold standard for inferring causality is the randomized controlled trial (RCT). Given the ethical constraints, RCTs are difficult and sometimes even infeasible to implement. Mendelian randomization analysis is superior to traditional observational studies for assessing the causal relationship between modifiable exposures or risk factors and clinically relevant outcomes (3–5) (Figure 1A). This approach avoids biased results caused by confounding or reverse causality to the greatest extent possible and allows for a large sample size to be studied. The sample size is expanded, thus, increasing the statistical validity of the study. These advantages are achieved by using genetic variation as an exposed instrumental variable. The wide availability of published genome-wide association analyses for screening out suitable genetic instrumental variables makes Mendelian randomization analysis a time-saving and low-cost method.

Available studies have identified multiple risk factors for epilepsy as well as causal mechanisms through a Mendelian randomization approach. Of these risk factors, improved educational attainment and cognitive function reduced the risk of epilepsy (6). Alcohol intake may contribute to an elevated risk of epilepsy, whereas milk intake may be associated with a reduced risk of epilepsy, and coffee intake has no association with the risk of epilepsy (7). Therefore, we might be able to prevent the risk of developing epilepsy by increasing the number of years of education and changing lifestyles to prevent the development of epilepsy, which can help alleviate the burden of the disease. Furthermore, in terms of mechanism, a strong positive correlation was found between serum ferritin and transferrin saturation and the risk of epilepsy. Two articles on ferroptosis and its role in epilepsy, as well as epileptic iron metabolism and ferroptosis, laterally elucidated the relevance of iron to epilepsy in conjunction with the findings of Mendelian randomization study (8–10), which provides a direction for the discovery of new targets for epilepsy treatment.

Several animal studies have presented evidence of a probable association between obesity and epileptic seizures. Lipocalin-deficient mice fed a high-fat diet accumulate more fat, resulting in increased seizures and increased hippocampal neuronal death (11). Kang et al. found that obesity induced by a high-fat diet caused red alginate-treated mice to exhibit more IV or V seizures behaviorally, a higher number and longer duration of seizure spike sequences on EEG, and pathologically exacerbated hippocampal neuronal death (12). While a correlation between obesity and epilepsy has been found in animal studies; only weight has been used as an indicator of obesity. There are limited studies that use humans as observational subjects, and observational studies have found that newly diagnosed and untreated children with epilepsy have a higher body mass index (BMI) (13, 14). A higher BMI may be associated with an increased risk of refractory epilepsy (15). However, the presence of factors such as epilepsy that affect hormone levels, the use of antiseizure medications, dietary differences, exercise, socioeconomic, and other factors may lead to the risk of reverse causality and confounding bias. Furthermore, only weight and BMI were used as indicators of obesity and fat distribution was ignored. It has long been documented that the same overweight or obese individuals with the same adiposity may pose quite different disease risks (16). BMI represents the standard for generalized obesity status, while waist circumference (WC) and waist-hip ratio (WHR) indicate visceral obesity (17) and there are multiple subtypes of epilepsy. Therefore, to date, the causal relationship between obesity and epilepsy has been unclear.

Therefore, we performed a Mendelian randomization analysis using data from genome-wide association studies on four indicators of waist circumference, hip circumference, waist-hip ratio, and BMI-with several epilepsies to further elaborate on the extent to which different epilepsy subtypes are affected by obesity and which obesity traits best explain the risk with epilepsy subtypes, which in turn help to deliver more effective nutritional interventions.

## Methods

### Experimental design

We conducted separate two-sample Mendelian randomization studies for the four indicators of obesity and epilepsy and its seven subtypes, in order to explore whether obesity was a risk factor for epilepsy. Subsequently, reverse Mendelian randomization and multivariate Mendelian randomization studies were carried out on significant outcomes, which were used to correct for the independent positive causality of the outcomes we found.

### Acquisition of summary statistics from genome-wide association analysis

Summary statistics of GWAS were obtained from the International League Against Epilepsy (ILAE) consortium cohort for all epilepsy (15,212 cases and 29,677 controls), hereditary generalized epilepsy (3,769 cases and 29,677 controls) and focal epilepsy (9,671 cases and 29,677 controls). Seven subphenotypes of epilepsy were also available: juvenile myoclonic epilepsy (JME), juvenile absence epilepsy (JAE), childhood absence epilepsy (CAE), focal epilepsy (documented hippocampal sclerosis), focal epilepsy (documented lesion negative), focal epilepsy (documented lesion other than hippocampal sclerosis), generalized epilepsy with tonic-clonic seizures. (18). The obesity-related indicators we selected were waist circumference, hip circumference, waist-to-hip ratio, and BMI. Waist circumference, hip circumference, and waist-hip ratio came from a genome-wide association study involving 224,459 people, (19) while the GWAS summary statics of BMI were obtained from a genome-wide association study of European height and body mass index with the largest sample size to date (20). As our epilepsy data are meta-data from a different ethnic group; no particular restrictions are made on ethnicity in our study. For the validation cohort, we changed the source of the waist-hip circumference and BMI data and used the results of a genome-wide association study conducted in the UKB population that did not overlap with the study cohort sample used by Loic Yengo et al.

### Two-sample Mendelian randomization

Two-sample Mendelian randomization was achieved using the package R of Two Sample MR (version 0.5.6), and we conducted a Mendelian randomization analysis using three models: inverse variance weighted (IVW), MR Egger, and weighted median. To ensure their validity, the selected instrumental variables should meet three key principles: (1) the instrumental variables were associated with indicators (*p* < 5e-8), (2) there was no association between the instrumental variables and epilepsy, and (3) the instrumental variables were not associated with confounders. The selected instrumental variables were also subsequently removed for imbalance SNPs (clump = 500 kb, *r*^2^ = 0.3).

### Reverse Mendelian randomization

The reverse Mendelian randomization study aims to investigate whether there is also reverse facilitation of the causal relationship between obesity and epilepsy. In this part of the study, we paired exposure and outcome of the significant causal pairs obtained in the previous step with other epilepsy or its subtypes as exposure and obesity and its associated indicators as an outcome. The instrumental variables were selected as above.

### Multivariate Mendelian randomization

In the course of the Mendelian randomization study, despite strict restrictions on the selection of instrumental variables, it was impossible to avoid the fact that the instrumental variables we selected were still somewhat pleiotropic. Some of the instrumental variables, such as those related to body mass index, will also be correlated with waist or hip circumference, so we cannot be sure. This approach has been used in multiple studies, for example, in an investigation of risk factors for gallstone disease, where the authors performed multivariate Mendelian randomization between LDL, HDL, and total cholesterol and gallstone disease (21) and ultimately found that lower levels of total cholesterol were an independent risk factor for gallstone disease. Therefore, in this step, we took the concurrent set of waist circumference, hip circumference, and obesity-related SNPs as instrumental variables (clump = 500 kb, *r*^2^ = 0.3) and performed a multivariate Mendelian randomization study for epilepsy, equivalent to separately correcting for the effect of a single exposure covariate.

### Statistical methods

In two-sample Mendelian randomization, we will define a result as statistically significant when the *p*-value for the IVW method is <0.05, and the *p*-value for either of the other two methods is <0.05. For sensitivity analysis of the results, we used Cochran's Q test to test for heterogeneity of the results and used the intercept term of the regression in the MR Egger method to indicate whether there was a horizontal multiplicity of the results. The statistical validity of the MR results was calculated on the mRND website (https://shiny.cnsgenomics.com/mRnd/), where the F statistic indicates the strength of the instrumental variable and can be calculated using the following formula.


$$\begin{array}{l} \text{F}\;={\text{R}}^{2}\left(\text{N}-2\right)\left(1-{\text{R}}^{2}\right) \end{array}$$

In this formula, N denotes the sample size and R2 denotes the variance of the phenotypes explained by the instrumental variables in the general population, and if there are <10 instrumental variables, R2 can be calculated using the following formula:


$$\begin{array}{l} {\text{R}}^{2}=\;2\times \text{EAF}\times \left(1-\text{EAF}\right)\times {\text{BETA}}^{2} \end{array}$$

If the instrumental variables are >10, then R2 can be calculated as follows:


$$\begin{array}{l} R^{2}=\frac{2\times EAF\times (1-EAF)\times BETA^{2}}{2\times EAF\times (1-EAF)\times N\times SE\times BETA^{2}}\\ \;+2\times EAF\times (1-EAF)\times BETA^{2} \end{array}$$

EAF indicates the allele frequency of the instrumental variable effect, beta is the effect size of the instrumental variable on exposure, se is the standard error of the instrumental variable on exposure, and *N* is the sample size of exposure. It should be noted that this method can only approximate the degree of explanation of the instrumental variable for exposure, as we do not have genotype data.

## Results

### Two-sample Mendelian randomization reveals obesity as a risk factor for epilepsy

**Figure 2:**
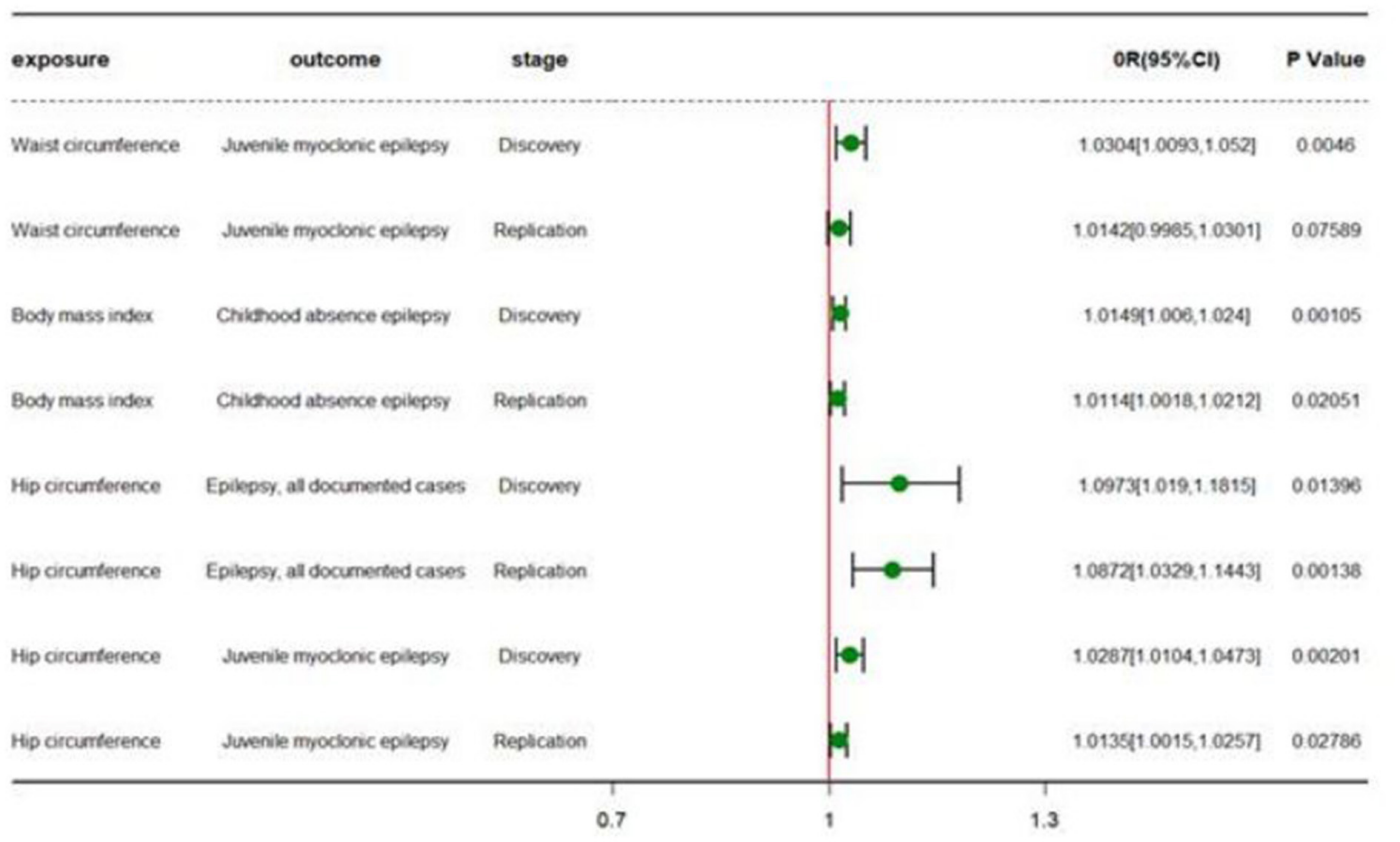
Forest plot of different TSMR stages. OR (95%CI) is the odds ratio of each MR estimate. *P*-value is the significant level of each MR estimate.

In the discovery set, we conducted a two-sample Mendelian randomization analysis of four quantitative indicators of obesity and epilepsy and its subtypes. The results found that waist circumference was a risk factor for juvenile myoclonic epilepsy (beta = 0.0299, *P* = 4.60 × 10^−3^) and that an increase in hip circumference increased the risk of juvenile myoclonic epilepsy and epilepsy with effect values of 0.0283 (*P* = 2.01 × 10^−3^) and 0.0928 (*P* = 1.40 × 10^−2^), respectively. Furthermore, children with a higher BMI had a higher risk of epilepsy (beta = 0.0148, *P* = 1.05 × 10^−3^) [Table 1: TSMR results forward and reverse TSMR results; Figure 2: forest plot (forward and reverse)].

**Table 1:** Stage indicates the stage of our MR study, which can be divided into the discovery set, the validation set, and reverse MR; exp indicates the exposure in the MR study; out indicates the outcome in MR study; nsnp indicates the number of instrumental variables used; method indicates the meta method used, which can be divided into IVW, MR Egger, and Weighted median; beta refers to the effect size in the MR analysis; se exhibits the standard error in MR analysis; p indicates the statistical significance in the MR analysis; Q indicates the significance of the heterogeneity test in the MR analysis; Ple indicates the significance level of the multiplicity test in the MR analysis; r^2^ indicates the degree of explanation of the instrumental variable for the exposure, with larger values indicating a greater degree of explanation for the exposure; f indicates the F-test statistic, with larger values indicating that the instrumental variable used in the MR analysis is a strong instrumental variable.

**Stage**	**Exposure**	**Outcome**	**Nsnp**	**Method**	**Beta**	**se**	**Pval**	**Method**	**beta**	**se**	**Pval**	**Method**	**Beta**	**Se**	**pval**	**Q**	**Ple**	**R2**	**F**	**MRpower**
Discovery	Wais circumference	juvenile myoclonic epilepsy || id:ieu-b-17	58	Inverse variance weighted (fixed effects)	0.0299	0.0106	4.60E-03	MR Egger	−0.0266	0.0460	0.5656	weighted	0.0451	0.0157	0.0041	0.2576	0.2112	0.0635	14601.27	1
Discovery	Hip circumference	Epilepsy, all documented cases || id:ieu-b-8	76	Inverse variance weighted (multiplicative random effects)	0.0928	0.0378	1.40E-02	MR Egger	0.0746	0.1586	0.6393	weighted	0.1300	0.0495	0.0088	0.0198	0.9063	0.0830	17150.02	1
Discovery	Hip circumference	juvenile myoclonic epilepsy || id:ieu-b-17	80	Inverse variance weighted (multiplicative random effects)	0.0283	0.0092	2.01E-03	MR Egger	0.0183	0.0384	0.6352	weighted	0.0306	0.0118	0.0096	0.0443	0.7898	0.0878	18047.95	1
Discovery	body mass index	childhood absence epilepsy || id:ieu-b-13	902	Inverse variance weighted (multiplicative random effects)	0.0148	0.0045	1.05E-03	MR Egger	0.0235	0.0132	0.0760	weighted	0.0149	0.0073	0.0428	1.51E-09	0.4845	0.5792	193906.01	1
Replication	Hip circumference	juvenile myoclonic epilepsy || id:ieu-b-17	551	Inverse variance weighted (multiplicative random effects)	0.0134	0.0061	2.79E-02	MR Egger	0.0164	0.0187	0.3822	weighted	0.0139	0.0095	0.1423	1.15E-06	0.8678	0.5132	115447.75	1
Replication	Hip circumference	epilepsy,all documented cases || id:ieu-b-8	536	Inverse variance weighted (multiplicative random effects)	0.0836	0.0261	1.38E-03	MR Egger	0.0453	0.0810	0.5759	weighted	0.0748	0.0368	0.0417	3.05E-12	0.6176	0.4989	115528.18	1
Replication	Body mass index (BMI)	childhood absence epilepsy || id:ieu-b-13	658	Inverse variance weighted (multiplicative random effects)	0.0114	0.0049	2.05E-02	MR Egger	0.0133	0.0146	0.3632	weighted	0.0176	0.0073	0.0154	4.84E-11	0.8883	0.6148	109285.90	1
Replication	Waist circumference	juvenile myoclonic epilepsy || id:ieu-b-17	449	Inverse variance weighted (multiplicative random effects)	0.0141	0.0079	7.59E-02	MR Egger	0.0201	0.0242	0.4071	weighted	0.0155	0.0131	0.2365	8.48E-08	0.7925	0.4704	115135.02	1
Reverse	Juvenile myoclonic epilepsy || id:ieu-b-17	Hip circumference	1	wald ratio	0.4830	0.3583	1.78E-01	NA	NA	NA	NA	NA	NA	NA	NA	NA	NA	7.52E-05	2.32	0.29
Reverse	juvenile myoclonic epilepsy || id:ieu-b-17	Waist circumference	1	wald ratio	0.7322	0.3427	3.26E-02	NA	NA	NA	NA	NA	NA	NA	NA	NA	NA	7.52E-05	2.32	0.88
Reverse	epilepsy, all documented cases || id:ieu-b-8	Hip circumference	3	Inverse variance weighted (multiplicative random effects)	−0.0203	0.0523	6.99E-01	NA	0.1294	0.1882	0.6164	weighted	−0.0059	0.0632	0.9262	0.4788	0.5600	0.0032	145.08	0.09
Reverse	childhood absence epilepsy || id:ieu-b-13	body mass index	1	wald ratio	0.8981	0.1815	7.52E-07	NA	NA	NA	NA	NA	NA	NA	NA	NA	NA	4.56E-05	1.39	**0.59**

A value of F below 10 generally indicates a strong instrumental variable, and the MR results might be biased due to the lack of strength of the instrumental variable.

### Validation in the UKB dataset

The results in the discovery set were then validated using the UKB queue population. We chose the GWAS study conducted by the MRC-IEU team on BMI, waist, and hip circumference as exposure and childhood absence epilepsy, juvenile myoclonic epilepsy, and epilepsy from the ILAE (International League Against Epilepsy Consortium on Complex Epilepsies) were used as outcome in another two-sample Mendelian randomization study. The results confirmed our findings. Namely, increased waist and hip circumference increased the risk of juvenile myoclonic epilepsy, with effect values of 0.0134 (*p* = 2.79 × 10^−2^) and 0.0141 (*p* = 7.59 × 10^−2^), respectively. High BMI also increased the risk of childhood absence epilepsy (beta = 0.0114, *P* = 2.05 × 10^−2^) (Table 1).

### Inverse effect of epilepsy on obesity in adolescents and children

To further investigate the causal relationship between obesity and epilepsy, we conducted a reverse Mendelian randomization study on the above results. The results showed that childhood absence epilepsy increased BMI (beta = 0.8980, *P* = 7.52 × 10^−7^), and juvenile myoclonic epilepsy increased waist circumference (beta = 0.7322, *P* = 3.26 × 10^−2^) (Table 1).

### Multivariate Mendelian randomization uncovers independent risk factors

Due to the particularly strong correlation among the four obesity indicators of BMI (waist circumference, hip circumference, waist-hip ratio), which prevents us from choosing instrumental variables that are not horizontally pleiotropic, we then performed multivariate Mendelian randomization. Following correction for their interactions, we found that waist circumference, hip circumference, waist-hip ratio, and BMI were all independent of juvenile myoclonic epilepsy. Increases in hip circumference and waist-hip ratio increased the risk of juvenile myoclonic epilepsy, with effect values of 0.1051 (*P* = 9.75 × 10^−4^) and 0.1430 (*P* = 3.99 × 10^−3^), respectively, while increases in BMI and waist circumference conversely decreased their risk, with effect values of −0.0951 (*P* = 3.14 × 10^−2^) and −0.0541 (*P* = 1.71 × 10^−2^). On the contrary, the multivariate Mendelian randomization did not reveal independent risk factors for childhood absence epilepsy and epilepsy, which implies that the effect of obesity on childhood absence epilepsy is influenced by both increased BMI and interactions such as waist circumference and hip circumference (Supplementary Figures 1–3; Table 2).

**Table 2:** Exposure indicates the exposure in MVMR, outcome indicates the outcome in MVMR, nsnp indicates the number of instrumental variables, beta indicates the effect size of exposure on the outcome calculated in MVMR, Se indicates the standard error, and pval indicates the significance level of the MVMR analysis.

**Exposure**	**Outcome**	**Nsnp**	**b**	**se**	**P-value**
Hip circumference	juvenile myoclonic epilepsy	23	0.031880549	0.031880549	0.000974525
Waist circumference	juvenile myoclonic epilepsy	16	0.044176557	0.044176557	0.031387467
Waist-to-hip ratio	juvenile myoclonic epilepsy	10	0.049661782	0.049661782	0.003988294
body mass index	juvenile myoclonic epilepsy	416	0.022683526	0.022683526	0.017099335
Hip circumference || id:ieu-a-54	childhood absence epilepsy	23	0.025913189	0.025913189	0.225508844
Waist circumference || id:ieu-a-66	childhood absence epilepsy	16	0.035912169	0.035912169	0.348250882
Waist-to-hip ratio || id:ieu-a-72	childhood absence epilepsy	10	0.040372546	0.040372546	0.098085392
body mass index || id:ieu-b-40	childhood absence epilepsy	416	0.018440906	0.018440906	0.550131294
Hip circumference || id:ieu-a-54	epilepsy, all documented cases	22	0.136717486	0.136717486	0.70707981
Waist circumference || id:ieu-a-66	epilepsy, all documented cases	16	0.188782959	0.188782959	0.599696671
Waist-to-hip ratio || id:ieu-a-72	epilepsy, all documented cases	10	0.212886845	0.212886845	0.836123172
body mass index || id:ieu-b-40	epilepsy, all documented cases	401	0.097236479	0.097236479	0.465194552

## Discussion

To our knowledge, the present study provides the first illustration of the causal relationship between obesity and fat distribution and epilepsy subtypes using Mendelian randomization study. Obesity was identified as a risk factor for epilepsy by two-sample Mendelian randomization analysis, where the waist and hip circumference increased the risk of myoclonus in adolescents, and high BMI increased the risk of childhood absence epilepsy, followed by reverse Mendelian randomization and multivariate Mendelian randomization studies for significant results, which were used to calibrate our findings (Figure 1B). Taken together, these results suggested that an increase in hip circumference and waist-hip ratio could increase the risk of juvenile myoclonic epilepsy, with effect sizes of 0.1051 (*P* = 9.75 × 10^−4^) and 0.1430 (*P* = 3.99 × 10^−3^), respectively, while an increase in BMI and waist circumference would decrease the risk, with effect sizes of −0.0951 (*P* = 3.14 × 10^−2^) and −0.0541 (*P* = 1.71 × 10^−2^), respectively. (*P* = 1.71 × 10^−2^).

The study proposed that what was associated with different epileptic subtypes involved not only the volume of obesity but also the location of its distribution, which supported the emerging theory that the site of body fat deposition may play an essential role independent of total fat. Available evidence suggests that obesity can affect the risk of epilepsy through a variety of potential mechanisms. It is well known that obesity leads to an elevated inflammatory response and that excess adipose tissue increases the secretion of pro-inflammatory cytokines, markers of inflammation, including interleukin 6, interleukin 8, tumor necrosis factor, and C-reactive protein (22). Furthermore, inflammation impairs the neural circuits that control satiety, resulting in lipid abnormalities downstream, which leads to a vicious circle (23). Clinical and animal studies have shown a complex role of inflammation in the development and progression of epilepsy (24). Furthermore, neuroimaging studies have shown that obesity is accompanied by focal structural changes in many regions of the brain (25, 26) and a large prospective study over an eight-year period revealed an association between overweight and atrophy of the hippocampal volume (27). Meanwhile, hippocampal atrophy is the pathogenesis of drug-resistant epilepsy (28). High BMI increases the risk of childhood epilepsy, which is consistent with a previous retrospective study on the comorbidity of obesity in childhood epilepsy (14). The precise mechanisms of obesity and epilepsy remain to be further investigated.

There are limitations to our study. Firstly, While we have used various MR methods to avoid confusion caused by pleiotropy, we cannot completely rule out residual bias, which is an inherent limitation of MR methods. In addition, our findings were limited to individuals of European ancestry. Future replication of our findings in different races is needed for validation. Furthermore, it was not likely to determine the sex- as well as age-specific effects of obesity on epilepsy because individual patient data were not available in the GWAS dataset.

In summary, the available results imply that whatever the exact mechanism by which obesity increases the tendency and incidence of seizures, early prevention and treatment are apparently required to ensure long-term weight management.

## Author contributions

KZ wrote the manuscript and prepared data. HY and RC modified the manuscript. WW and ZQ designed the manuscript. All authors contributed to the article and approved the submitted version.

## Conflict of interest

The authors declare that the research was conducted in the absence of any commercial or financial relationships that could be construed as a potential conflict of interest.

## Publisher's note

All claims expressed in this article are solely those of the authors and do not necessarily represent those of their affiliated organizations, or those of the publisher, the editors and the reviewers. Any product that may be evaluated in this article, or claim that may be made by its manufacturer, is not guaranteed or endorsed by the publisher.
