# Supplementary material for: Causal relationship among obesity and body fat distribution and epilepsy subtypes
Source: Front Neurol. 2022 Oct 26;13:984824. doi: 10.3389/fneur.2022.984824 (PMC9644162; doi:10.3389/fneur.2022.984824)
Supplement: Supplementary file 1 [file Data_Sheet_1.docx]

Supplementary Material

## Supplementary Figures

## MVMR results

## Epilepsy. all documented cases


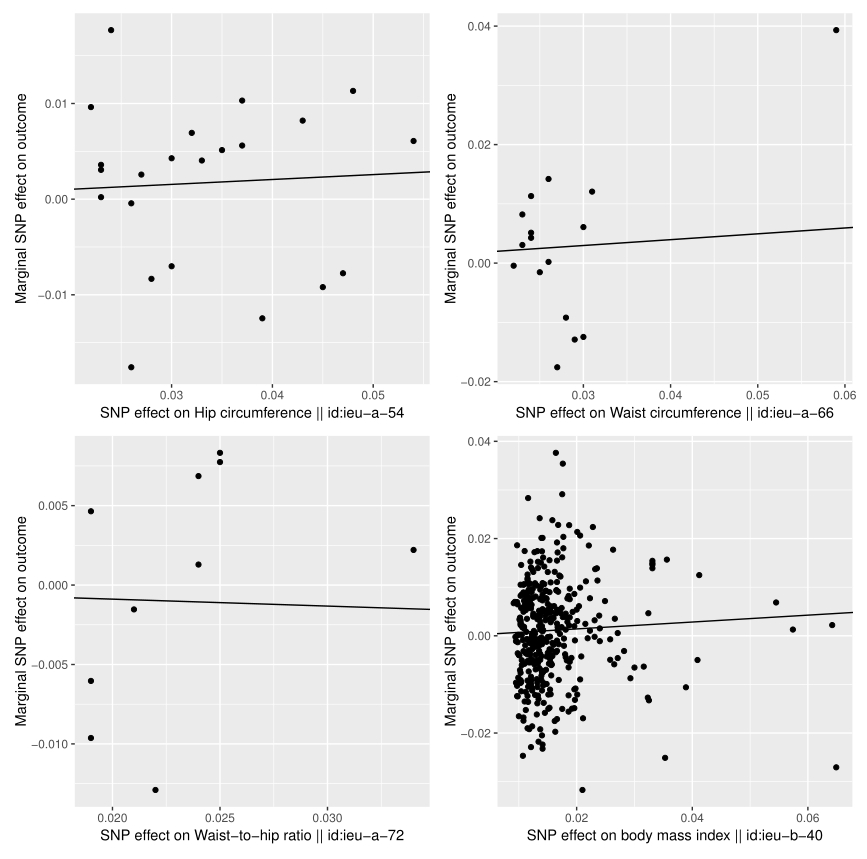


**Supplementary Figure 1.** Multivariate MR scatter plot of obesity on epilepsy.

MVMR results

Childhood absence epilepsy


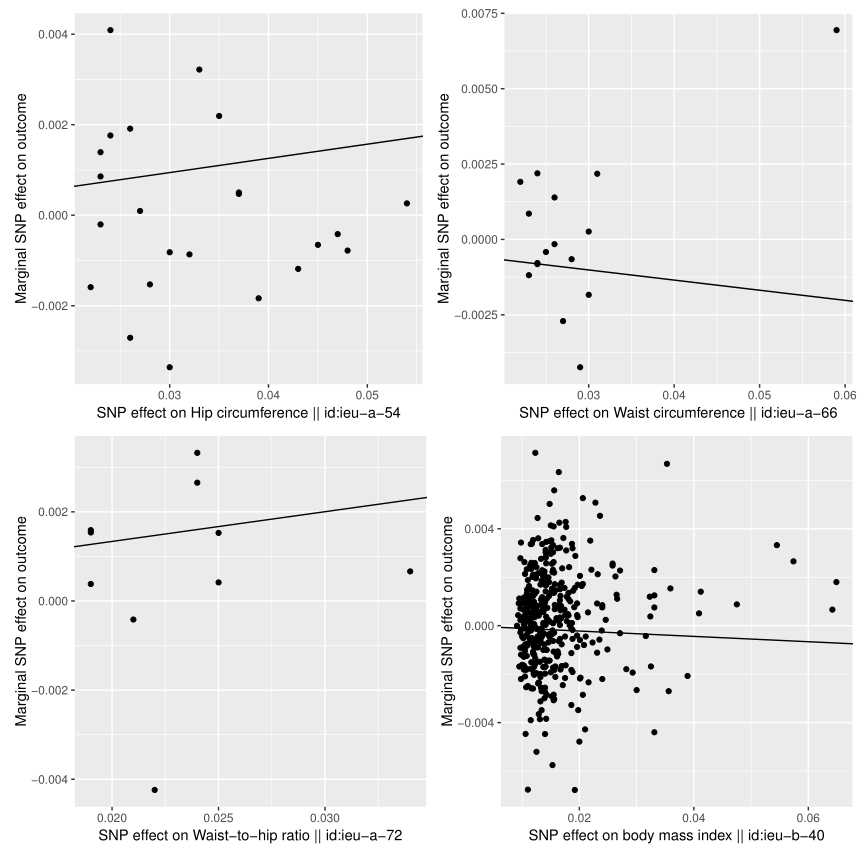


**Supplementary Figure 2.** Multivariate MR scatter plot of obesity on childhood absence epilepsy.

MVMR results

Juvenile myoclonic epilepsy


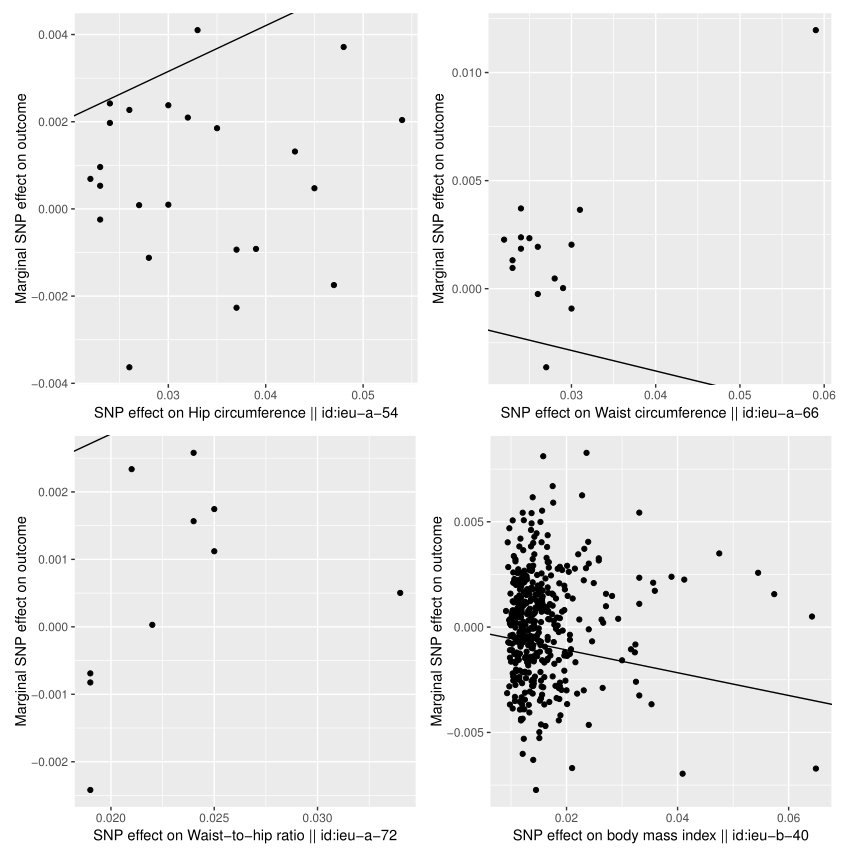


**Supplementary Figure 3.** Multivariate MR scatter plot of obesity on juvenile myoclonic epilepsy.
